# Effects of Serum C-Peptide Level on Blood Lipid and Cardiovascular and Cerebrovascular Injury in Patients with Type 2 Diabetes Mellitus: A Meta-Analysis

**DOI:** 10.1155/2022/6314435

**Published:** 2022-04-08

**Authors:** Juan Qin, Rongli Sun, Ding Ding

**Affiliations:** ^1^Department of Endocrinology and Metabolism, The Fourth People's Hospital of Shenyang, Shenyang 110031, Liaoning, China; ^2^Center for Clinical Biological Samples, The First Affiliated Hospital of Jinzhou Medical University, Jinzhou 121000, Liaoning, China; ^3^Department of Clinical Nutrition, Shengjing Hospital of China Medical University, Shenyang 110000, Liaoning, China

## Abstract

**Objective:**

This study aims to investigate the effects of serum C-peptide levels on blood lipid and cardiovascular and cerebrovascular injury in patients with type 2 diabetes mellitus (T2DM).

**Methods:**

China National Knowledge Infrastructure (CNKI), WanFang Data, PubMed, Web of Science, and Embase databases were searched for relevant studies published from January 2010 to June 2021. All retrieved randomized controlled trials that evaluated the effect of serum C-peptide levels on blood lipids or cardiovascular and cerebrovascular injuries in T2DM patients were included in our study. Patients in the included studies were divided into normal C-peptide group (control group) and low C-peptide group (treatment group) according to fasting C-peptide levels. Meta-analysis was performed using Stata16.0.

**Results:**

A total of 7 studies were included for the meta-analysis. Compared with the control group, the treatment group was associated with a higher incidence of coronary heart disease (OR = 4.89; 95% CI: 1.13, 21.24; *P* < 0.05) and cerebral infarction (OR = 3.24; 95% CI: 0.59, 17.66; *P* < 0.05). In addition, patients in the treatment group had significantly higher levels of total cholesterol (SMD = 0.01; 95% CI: −0.38, 0.39; *P* < 0.05), triglyceride (SMD = 0.62; 95% CI: 0.24, 1.00; *P* < 0.05), glycated hemoglobin (SMD = 0.25; 95% CI: −0.50, 1.00; *P* < 0.05), and low-density lipoprotein cholesterol (SMD = 0.23; 95% CI: −0.00, 0.46; *P* < 0.05). However, there was no significant difference in high-density lipoprotein cholesterol levels between the two groups (SMD = 0.30; 95% CI: -0.26, 0.86; *P* > 0.05).

**Conclusions:**

Low serum C-peptide level significantly increases the incidence of coronary heart disease and cerebral infarction. Additionally, low serum C-peptide increases blood lipid level and promotes lipid deposition. Collectively, low serum C-peptide has a negative impact on the occurrence and development of T2DM and therefore serum C-peptide level needs to be adjusted timely.

## 1. Introduction

Diabetes mellitus (DM) is a group of endocrine and metabolic disorders, characterized by hyperglycemia due to insufficient insulin secretion or insulin action. It is one of the major chronic diseases that seriously threaten human health. Its incidence is second only to cardiovascular and cerebrovascular diseases and tumors. The prevalence of DM has reached 9.7% in China [1]. In general, this disease has become a common and frequently-occurring disease nowadays, and its complications seriously affect the quality of life of patients and even threaten life [2, 3]. Among DM, type 2 DM (T2DM) is the most common metabolic disease, characterized by elevated blood glucose and along with other chronic diseases such as hypertension, atherosclerosis, and hyperlipidemia [4]. The main cause of death in T2DM is cardiovascular disease [5].

C-peptide is a 31-amino acid polypeptide and is a cleavage product of proinsulin, acting as a more accurate marker with higher stability than insulin [6]. Therefore, C-peptide measurement is generally used clinically to assess islet function in diabetic patients [7]. Recently, it has been pointed out that C-peptide can cause atherosclerotic lesions in T2DM patients [8]. Liu et al. [9] reported that 2-h serum C-peptide level was found to be a risk factor for changes in carotid intima-media thickness. Chung et al. [10] also showed a negative correlation between serum C-peptide level and cardiovascular autonomic neuropathy in T2DM patients. Collectively, these studies all demonstrated a close relationship between C-peptide level and T2DM complications. However, there is a lack of systematic research on the effects of C-peptide level on blood lipids as well as cardiovascular and cerebrovascular diseases in T2DM patients. Therefore, this meta-analysis aims to systematically investigate and evaluate the correlation between different C-peptide levels and T2DM complications. The conclusion of this study is expected to provide further research ideas for the diagnosis, treatment, and prognosis of diabetic patients.

## 2. Materials and Methods

### 2.1. Literature Retrieval

Systematic searches were performed in the China National Knowledge Infrastructure (CNKI), WanFang Data, PubMed, Web of Science, and Embase databases to obtain relevant studies published from January 2010 to June 2021. The search syntax was (“serum C-peptide”) AND (“type 2 diabetes mellitus”) AND (“blood lipid”) AND/OR (“cardiovascular and cerebrovascular”).

### 2.2. Screening Criteria

Studies conforming to the inclusion criteria were selected: population: patients diagnosed with T2DM; intervention: patients with low C-peptide level (treatment group); comparison: patients with normal C-peptide level (control group); outcomes: incidence of cerebral infarction and coronary heart disease, total cholesterol (TC), triglyceride (TG), high-density lipoprotein cholesterol (HDL-C), low-density lipoprotein cholesterol (LDL-C), glycated hemoglobin (HbAlc); studies: randomized controlled trial studies.

The exclusion criteria were (1) patients diagnosed as non-T2DM; (2) literature with missing data, duplicate reports; and (3) nonrandomized controlled trial, letter, review, case report.

### 2.3. Literature Data Extraction

Two reviewers independently screen the literature and extract the data according to the inclusion and exclusion criteria. The following data were collected from the selected studies: (1) baseline characteristics of the included studies; (2) baseline characteristics of patients; (3) sample size; (4) study design; (5) study results. When opinions differed, the two reviewers discussed or a third review assisted the two to reach consensus. If necessary, authors of the selected studies were contracted to consult data not mentioned in the articles. For duplicate reports or extending reports, the ones that had complete data or were published recently were selected.

### 2.4. Statistical Analysis

Statistical analysis was performed using Stata16.0 software (StataCorp LLC, College Station, TX, USA). Firstly, *χ*2 test was used to test the heterogeneity among the including literature, with *α* = 0.05 as a significance level. The fixed effects model was adopted for pooling effect sizes if I^2^ < 50% and *P* > 0.10; otherwise, the random effects model was used. Standard mean difference (SMD) was used as a statistic for measurement data, while odds ratio (OR) and 95% confidence interval (CI) for categorical variables. The difference was statistically significant when *P* < 0.05.

## 3. Results

### 3.1. Literature Retrieval Results

**Figure 1:**
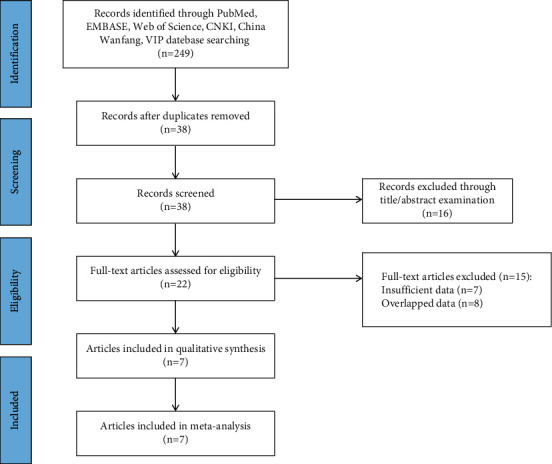
Flow diagram of study selection.

According to the search syntax, 249 articles were preliminarily obtained. Then, we excluded 211 duplicate articles, 16 case reports, and 15 noneligible articles (7 with insufficient data, 8 with duplicate data). Finally, 7 studies were included in the meta-analysis [11–17]. A total of 2588 patients with T2DM were included, of which 1735 were in the control group; 853 were in the low C-peptide group. The literature screening process is shown in Figure 1. Characteristics of each included study are shown in Table 1.

### 3.2. Meta-Analysis Results

#### 3.2.1. Incidence of Cardiovascular and Cerebrovascular Injuries in Patients with Type 2 Diabetes Mellitus (T2DM)

**Figure 2:**
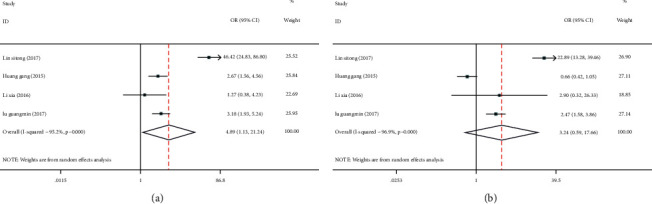
Meta-analysis of the effect of serum C-peptide level on cardiovascular and cerebrovascular injuries in patients with type 2 diabetes mellitus (T2DM). (a) Forest plot of the incidence of coronary heart disease. (b) Forest plot of the incidence of cerebral infarction.

Four articles [12, 13, 15, 16] reported cardiovascular and cerebrovascular injuries (incidence of coronary heart disease and cerebral infarction). There was heterogeneity among the studies regarding coronary heart disease (I^2^ = 95.2%, *P* ≤ 0.001) and cerebral infarction (I^2^ = 96.9%, *P* ≤ 0.001), so the random effects model was used to pool effect sizes. The results showed that the incidences of coronary heart disease (OR = 4.89; 95% CI: 1.13, 21.24; *P* < 0.05) and cerebral infarction (OR = 3.24; 95% CI: 0.59, 17.66; *P* < 0.05) in the low C-peptide group were significantly higher than those in the control group, Figures 2(a) and 2(b).

**Figure 3:**
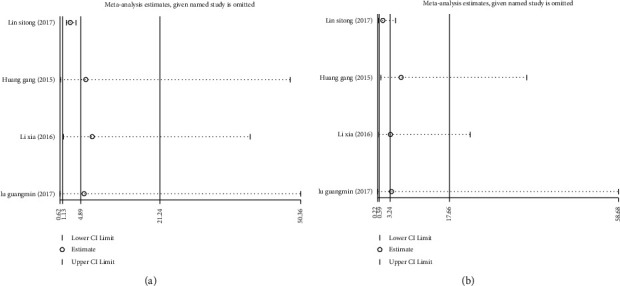
Sensitivity analysis of the effect of serum C-peptide level on cardiovascular and cerebrovascular injuries in patients with type 2 diabetes mellitus (T2DM). (a) Sensitivity analysis of the incidence of coronary heart disease. (b) Sensitivity analysis of the incidence of cerebral infarction.

Due to heterogeneity among the included studies, sensitivity analyses were required. Using one by one elimination, we found that the literature by Lin (2017) [12] might be the main source of increased heterogeneity. After excluding this literature, the obtained results still showed that the incidence of coronary heart disease, Figure 3(a), and cerebral infarction, Figure 3(b), in the low C-peptide group was higher than that in the control group. Therefore, the above results were relatively stable and reliable.

#### 3.2.2. Meta-Analysis of Serum Lipid Parameters in Patients with Type 2 Diabetes Mellitus (T2DM)

**Figure 4:**
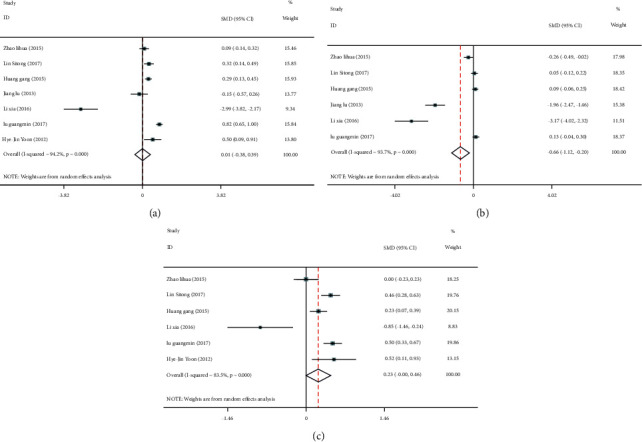
Forest plots of the relationship between serum C-peptide levels and serum total cholesterol TC; (a) triglyceride (TG) (b) and glycosylated hemoglobin (HbAlc) (c) levels in patients with type 2 diabetes mellitus (T2DM).

Seven studies [11–17] reported the effect of different serum C-peptide levels on TC, 6 articles [11–16] reported its effect on TG, and 6 articles [11–15, 17] reported it on HbAlc. Significant heterogeneity was identified among the included studies (I^2^ > 50%), so the random effects model was used to pool effect sizes. The results showed that TC level (SMD = 0.01; 95% CI: -0.38, 0.39; *P* < 0.05) (Figure 4(a)), TG level (SMD = 0.62; 95% CI: 0.24, 1.00; *P* < 0.05) (Figure 4(b)), and HbAlc level (SMD = 0.25; 95% CI: -0.50, 1.00; *P* < 0.05) (Figure 4(c)) were significantly higher in the low C-peptide group than in the control group.

**Figure 5:**
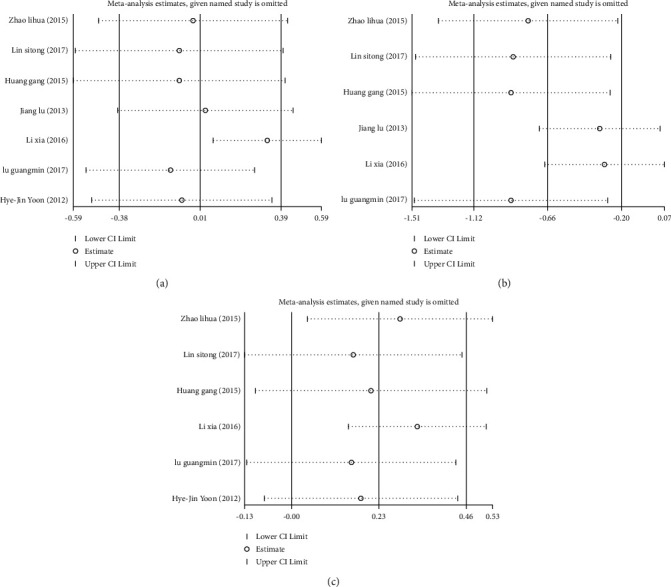
Sensitivity analysis of the relationship between serum C-peptide levels and serum total cholesterol TC; (a) triglyceride (TG) (b) and glycosylated hemoglobin (HbAlc) (c) levels in patients with type 2 diabetes mellitus (T2DM).

Sensitivity analyses were required due to heterogeneity among the included studies. Through the one by one elimination method, it was found that these two literatures, Jiang and Ma (2013) [14] and Lixia (2016) [15], might be the main source of increased heterogeneity. After these two literatures were excluded, the obtained results were consistent with the forest plot results, confirming that the resulting data were robust and reliable, Figures 5(a)–5(c).

**Figure 6:**
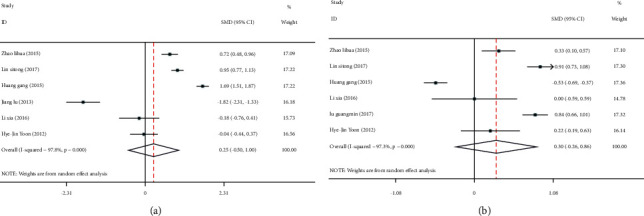
Forest plots of the effect of serum C-peptide levels on HDL-C (a) and LDL-C (b) levels in patients with type 2 diabetes mellitus (T2DM).

Additionally, six articles [11–13, 15–17] compared HDL-C and LDL-C levels between the two groups. Due to high heterogeneity among the included studies (HDL-C : I^2^ = 97.3%, *P* ≤ 0.001; LDL-C : I^2^ = 83.5%, *P* ≤ 0.001), the random effects model was adopted to pool effect sizes. The results showed no significant difference in HDL-C levels between the two groups (SMD = 0.30; 95% CI: -0.26, 0.86; *P* = 0.294), Figure 6(a). However, LDL-C levels were significantly higher in the low C-peptide group than in the control group (SMD = 0.23; 95% CI: -0.00, 0.46; *P* < 0.05), Figure 6(b).

**Figure 7:**
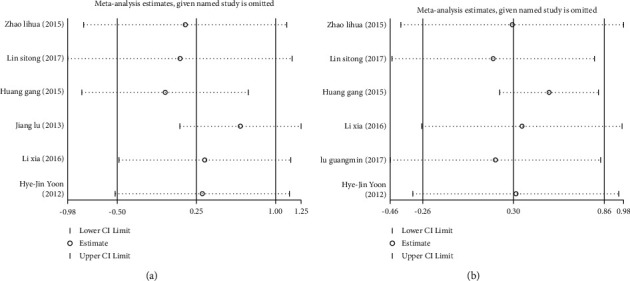
Sensitivity analyses of the effect of serum C-peptide levels on HDL-C (a) and LDL-C (b) levels in patients with type 2 diabetes mellitus (T2DM).

Further sensitivity analyses found that Huang Gang (2015) [13] and Li Xia (2016) [15] might be the main sources of increased heterogeneity in HDL-C and LDL-C, respectively. The results obtained after excluding these two literatures were consistent with the results before exclusion, Figures 7(a)–7(b). This meant that the above results were relatively stable and reliable.

## 4. Discussion

DM is one of the most common chronic diseases, which seriously affects the quality of life of patients. T2DM, a more severe type of DM, is more intractable to existing treatments and may cause a variety of complications, including renal failure, blindness, coronary heart disease, and cerebral infarction [18, 19]. Targeted treatment is therefore required. C-peptide has been proved to be an active peptide hormone with potentially important physiological effects [6]. C-peptide can stimulate Na^+^-K^+^-ATPase and nitric oxide synthase activity [20, 21]. In type 1 DM patients lacking C-peptide, administration of this hormone can increase blood flow to skeletal muscle and skin, reduce glomerular hyperfiltration and urinary albumin excretion, and improve neurological function [7, 22]. In addition, studies have shown that C-peptide is beneficial for treating cardiovascular and cerebrovascular complications and controlling blood lipid in T2DM patients [23, 24].

Based on existing studies, in this study, we systematically assessed the effect of serum C-peptide levels on blood lipid as well as cardiovascular and cerebrovascular complications in the T2DM patients. A total of 7 studies were finally included in this meta-analysis. The results of the meta-analysis showed that low serum C-peptide was associated with higher incidence of cerebral infarction and coronary heart disease in T2DM patients, indicating serum C-peptide level as a cardiovascular and cerebrovascular risk factor [16].

Further, we analyzed the relationship between C-peptide levels and blood lipid levels in T2DM patients. The results revealed that, compared with the control group, the low C-peptide group showed higher levels of TC, TG, HbAlc, and LDL-C in T2DM patients. It is illustrated that low C-peptide has a definite effect on T2DM patients; patients with lower C-peptide levels are more likely to develop dyslipidemia [25]. Also, the results indicate that T2DM patients with low serum C-peptide level have a higher risk of atherosclerosis than those with normal serum C-peptide level; blood glucose levels are also generally higher in the former and may contribute to the development of macroangiopathy as well as coronary heart disease [26]. Therefore, low serum C-peptide is a risk factor that needs to be prevented. Additionally, previous studies have shown that low C-peptide may cause cardiovascular and cerebrovascular complications and hyperlipidemia complications in T2DM patients. For example, Huang et al. showed that TC and LDL-C levels were significantly higher in T2DM patients in the low C-peptide group than in the control group. They also found that low C-peptide caused an increased incidence of various complications including diabetic nephropathy, retinopathy, and coronary heart disease [13]. This is similar to our results, suggesting that C-peptide plays a vital role in microvascular complications of T2DM. At present, the mechanism by which low C-peptide leads to a high incidence of complications in T2DM patients is not very clear. Existing studies speculate that this mechanism is related to the fact that C-peptide can inhibit vascular smooth muscle cell proliferation and reduce the adverse effects of insulin on smooth muscle cells; and reduction of C-peptide level significantly affects the metabolism of blood lipids and blood glucose, thus easily leading to a higher incidence of complications [27, 28].

Since this study is a secondary research whose quality mainly depends on the quality of original research, this meta-analysis may have some limitations: (1) this study mainly collects relevant literature by searching electronic databases, and the references within the included studies are manually reviewed. Manual screening of the included references is done. Therefore, omission may occur due to possible shortcomings in terms of literature offered by databases and retrieval strategy; (2) most included studies are domestic ones. Based on the quality assessment of clinical randomized controlled trials, the comprehensive evaluation of the quality of included studies is not very high. There may be resulting bias in statistical results; (3) this meta-analysis only includes seven studies, and the sample size is small. That results in no significant difference in HDL-C levels between the two groups and insufficient statistical power. Therefore, we also need to carry out a well-designed, large-sample, multicenter, prospective clinical study for verifying our results in order to improve clinical treatment.

## 5. Conclusions

In summary, low C-peptide levels will accelerate the occurrence of cardiovascular and cerebrovascular diseases such as coronary heart disease and cerebral infarction in T2DM patients. Also, C-peptide affects the blood lipid levels of patients. Therefore, serum C-peptide levels should be strictly controlled to prevent complications in T2DM patients.

## Figures and Tables

**Table 1:** Basic characteristics of included studies.

Study	Sample time (year.month)	Cases Treat/Con	Age (years)		Gender ratio (male/female)		Disease course		Study design	Outcome measures
			Treat group	Con group	Treat group	Con group	Treat group	Con group		
Zhao, 2015 [11]	2013.06–2014.01	147/142	55.3 ± 12.5	58.3 ± 11.2	93/54	85/57	9.9 ± 8.8	7.7 ± 6.4	Retrospective	③④⑤⑥⑦
Lin, 2017 [12]	2012.03–2013.04	180/454	60.2 ± 10.7	59.7 ± 11.4	86/94	218/233	6.7 ± 5.8	6.5 ± 6.0	Retrospective	①②③④⑤⑥⑦
Huang, 2015 [13]	2011.03–2013.09	215/543	60.3 ± 12.5	60.1 ± 10.5	123/92	270/273	6.9 ± 1.1	6.5 ± 1.1	Retrospective	①②③④⑤⑥⑦
Jiang, 2013 [14]	2010.08–2012.11	45/45	58.3 ± 6.74	61.1 ± 6.7	40/5	38/7	4.4 ± 2.7	12.0 ± 4.0	Retrospective	③④⑦
Li, 2016 [15]	2013.03–2014.03	37/16	59.1 ± 1.6	61.3 ± 2.95	22/15	8/8	7.1 ± 1.1	6.7 ± 0.8	Retrospective	①②③④⑤⑥⑦
Lu, 2017 [16]	2014.01–2016.09	189/478	59.7 ± 4.5	60.1 ± 4.7	90/99	233/245	6.3 ± 2.1	6.4 ± 2.3	Retrospective	①②③④⑤⑥
Yoon, 2012 [17]	2008.01–2009.12	40/57	58.9 ± 9.3	59.4 ± 8.3	NR	NR	10.2 ± 9.4	9.0 ± 7.7	Retrospective	③⑤⑥⑦

*Note*. Treat: treatment group; Con: control group. ① Incidence of coronary heart disease. ② Incidence of cerebral infarction. ③ Total cholesterol. ④ Triglycerides. ⑤ High-density lipoprotein cholesterol. ⑥ Low-density lipoprotein cholesterol. ⑦ Hemoglobin A1c.

## Data Availability

The data used to support the findings of this study are available from the corresponding author upon request.
